# CD4 T Follicular Helper Cells Prevent Depletion of Follicular B Cells in Response to Cecal Ligation and Puncture

**DOI:** 10.3389/fimmu.2020.01946

**Published:** 2020-08-12

**Authors:** Matthew D. Taylor, Mariana R. Brewer, Ana Nedeljkovic-Kurepa, Yihe Yang, Kalpana S. Reddy, Mabel N. Abraham, Betsy J. Barnes, Clifford S. Deutschman

**Affiliations:** ^1^The Division of Critical Care Medicine, Cohen Children’s Medical Center, Northwell Health, New Hyde Park, NY, United States; ^2^Department of Pediatrics, Zucker School of Medicine at Hofstra/Northwell, Hempstead, NY, United States; ^3^Sepsis Research Lab, The Feinstein Institutes for Medical Research, Manhasset, NY, United States; ^4^The Department of Pathology, Zucker School of Medicine at Hofstra/Northwell, Hempstead, NY, United States; ^5^Institute of Molecular Medicine, Feinstein Institutes for Medical Research, Manhasset, NY, United States

**Keywords:** cecal ligation and puncture, sepsis, long-term effects, B cells, T follicular helper cells, CD4 T cells, T cell memory, adaptive immunity

## Abstract

Recent studies have demonstrated that induction of a diverse repertoire of memory T cells (“immune education”) affects responses to murine cecal ligation and puncture (CLP), the most widely – used animal model of sepsis. Among the documented effects of immune education on CLP are changes in T cell, macrophage and neutrophil activity, more pronounced organ dysfunction and reduced survival. Little is known, however, about the effects of CLP on B cell responses, and how these responses might be altered by immune education. Importantly, effective B cell responses are modulated by IL21 produced by CD4^+^/CXCR5^+^/PD1^+^ T follicular helper (Tfh) cells. We examined the B cell population in control and immune educated mice 24 h and 60 days after CLP. Education alone increased Tfh cells. Twenty-four hours after CLP, Tfh cells were depleted. However, this reduction was less pronounced in immune educated mice than in controls and the percentage of CD4 T cells expressing a Tfh phenotype increased in the animals. CLP did not alter splenic architecture and decreased numbers of follicular, marginal, and germinal center B cells. CLP induced changes were not, however, noted following CLP in immune educated mice. At 60 days post – CLP, numbers of follicular, germinal center and marginal zone B cells were increased; this increase was more pronounced in immune educated mice. Finally, while CLP reduced the induction of antigen specific B cells in controls, this response was maintained following CLP in immune educated mice. Our data suggest that preexisting Tfh assists in rescuing the B cell response to CLP.

## Introduction

In contrast to other aspects of the immune system, study of the B cell response to sepsis has been limited. Previous studies have shown little beyond a progressive depletion of B cells over time (1) while more recent work has demonstrated that mortality from sepsis is associated with impaired B-cell maturation (2). Sepsis-induced depression of the adaptive immune response has been recognized for many years. However, a lack of data regarding the early B cell response represents an important gap in our understanding of this deadly disorder.

Investigation into the pathobiological underpinnings of sepsis have long relied on the use of animal models, most commonly cecal ligation and pucture (CLP) in mice and rats (3, 4). However, the use of this and other models of inflammatory disorders has been questioned based on a lack of correlation between genetic responses in mice and humans (5, 6). More recent studies on laboratory mice have identified immune deficiencies that may impact on their use as models of human disease. In contrast to patients and to pet store or “mice in the wild,” laboratory mice lack a memory T cell compartment. This deficiency likely reflects limited exposure to antigenic stimulation in the pathogen-free facilities where lab mice are reared and maintained (7, 8). Several approaches to address this concern have been developed. For example, Huggins, et al. have used co-housing of pathogen free mice with “pet store” mice to increase the number of TLR2^+^ and TLR4^+^ phagocytes prior to challenge with Listeria monocytogenes (9) while Sjaastad et al., immunized mice with an MHC-II-restricted peptide following CLP to examine T cell-dependent B cell activation following (10). Along the same lines, we have addressed the contribution of preexisting T cell memory in the mice by inducing widespread T cell memory via administration of an anti-CD3ε activating (11). This procedure, termed “immune education,” led to widespread increase in the numbers of CD4 and CD8 memory T cells. Additional experiments indicate that memory T cell expansion altered the response to CLP by enhancing innate immune responses, increasing organ dysfunction, and reducing survival (Taylor et al., unpublished data). In the experiments described here we detail the effects of immune education on B cell responses following CLP.

## Materials and Methods

### Mice

C57Bl/6J male mice were obtained from the Jackson Laboratory and maintained in the animal facility at the Feinstein Institute for Medical Research. All animal studies were approved by the Institutional Animal Care and Use Committee and adhered to National Institutes of Health and Animal Research: Reporting of *in vivo* Experiments (ARRIVE) guidelines.

### *In vivo* Immunization

A total of 50 μg of Ultra-LEAF Anti-mouse CD3ε Antibody (145-2C11, BioLegend, San Diego, CA, United States) and Ultra-LEAF isotype Armenian Hamster IgG control (HTK888, BioLegend) were administered to 11 week old mice through a retro-orbital venous sinus injection. Mice were then rested for 35 days to allow for T cell memory development and to ensure that no acute response remained. Details of the initial response to inoculation and of the T cell phenotype at 35 days following have been published separately (11). Briefly, anti-CD3ε treatment induces acute CD4 and CD8 T cell activation. The acute response resolves by day 5 following treatment. Initial inoculation causes an acute expansion of neutrophils, which resolves by 35 days post-treatment. Further, by 35 days following treatment, no acute effector CD4 or CD8 T cells remain, and there is an expansion of the CD4 central and effector memory T cell population and the effector memory CD8 T cell population in the spleen, liver, and lungs. The innate immune system is not altered at 35 days following anti-CD3ε treatment in unchallenged mice.

For antigen specific response experiments, 4-hydroxy-3-nitrophenylacetic acid (NP, 5 μg, Sigma Aldrich, St. Louis, MO, United States) was dissolved in PBS and injected into the peritoneum at the end of CLP surgery or into unoperated (T_0_) mice at the same time.

### Cecal Ligation and Puncture Procedure

Cecal ligation and puncture was performed on 16 week old mice under isoflurane anesthesia as previously described (12). Briefly, following exposure, the cecum was single ligated approximately 1cm from the tip and two 22-guage needle punctures performed in series. One millimeter of fecal content was expressed from the punctures. The incision was closed in layers and the mice were resuscitated with 50 mL/kg 0.9% NaCl. No antibiotics were given. Resuscitation was repeated at 24 and 48 h post-CLP/NP injection. Mice were euthanized at given time points after CLP with pentobarbital. The effects of organ dysfunction in this model parallel those noted in the Vienna Consensus Conference on Animal Models of Sepsis (4).

Historically, CLP as detailed in this work was associated with 50% mortality at 24 h. Further, when mice were examined with a clinical scoring system developed for CLP, educated mice appeared sicker than control mice (Supplementary Figure S1) (13).

### Leukocyte Isolation

Spleens were obtained from euthanized mice and immediately weighed. Sections were taken for hematoxylin and eosin stain. The remaining splenic tissue was homogenization and filtered at 70 μm. Red blood cells were lysed and cells counts were obtained using a Countess II Automated Cell Counter (Thermo Fisher Scientific, Waltham, MA, United States).

### Flow Cytometric Analysis

Immediately after suspension, cells were stained for flow cytometric analysis with LIVE/DEAD fixable viability dye (Life Technologies, Carlsbad, CA, United States) and the following antibodies: CD90.2, CD44, CD8a, CD4, CD62L, CD11a, CXCR5, PD1, CD69, B220, CD19, CD23, CD21/35, GL7, IgM, IgD, CD138, and CD93. NP-PE (Biosearch Technologies, Teddington, Middlesex, United Kingdom) was utilized to detect NP-specific cells. Full antibody details are available in Supplementary Table S1. All flow cytometric analysis was performed on a BD LSR Fortessa 16-color cell analyzer and analyzed using FlowJo software version 10 (BD Biosciences, San Jose, CA, United States). Gating Strategy for T cells is shown in Supplementary Figure S2 and for B cells is shown in Supplementary Figure S3.

### Cytokine Production Assays

To assess cytokine production in T cells, single cell suspensions were treated with anti-CD3 (5 μg/ml, Biolegend) and anti-CD28 (1.7 μg/ml, Biolegend) for 5 h in the presence of Brefeldin A (2 μg/ml, BD Biosciences, San Jose, CA, United States). An unstimulated control was analyzed alongside stimulation experiments to assess for background production (14).

### Statistical Analysis

Animal data were analyzed using Student’s two-tailed T test or using two-tailed analysis of variance with Dunnett’s correction where appropriate [Prism 7.0; GraphPad or SAS Studio University Edition (SAS)].

## Results

### CLP Differentially Depletes Splenic B Cell Subsets and Memory T Follicular Helper Cells

**FIGURE 1:**
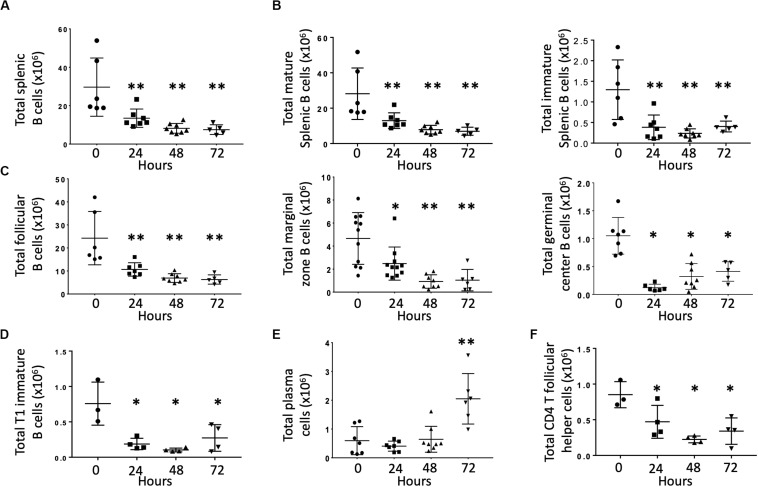
Effects of CLP on total B cells and B cell subtypes in the spleen. C57Bl/6 laboratory mice underwent CLP and were euthanized at given timepoints. Data obtained using flow cytometry. Unmanipulated mice were used as T_0_ controls. Each point represents results in an individual animal, central horizontal line indicates mean, upright, and inverted Ts indicate standard deviation, data representative of two independent experiments. *p < 0.05, **p < 0.01 for ** relative to T_0_ using one-way ANOVA with Dunnett *post hoc* correction for multiple comparisons. **(A)** Total splenic B cells at given time post-CLP. Gating: FSC/SSC, singlets, Live, CD19+/B220+; *N* = 4–6/group. **(B)** Total mature (left) and immature (right) splenic B cells at given time post-CLP. Gating: FSC/SSC, singlets, Live, CD19+/B220+, CD93; *N* = 4–6/group. **(C)** Total follicular, marginal zone, and germinal center B cells per spleen at given time post-CLP. Gating: Follicular B cells: FSC/SSC, singlets, Live, CD19^+^/B220^+^, CD93^–^, B220^+^/CD138^–^, IgM^lo^/CD21/35^lo^; Germinal center: FSC/SSC, singlets, Live, CD19^+^/B220^+^, CD93^–^, B220^+^/CD138^–^, IgM^lo^/CD21/35^lo^, GL7^+^; Marginal zone: FSC/SSC, singlets, Live, CD19^+^/B220^+^, CD93^–^, B220^+^/CD138^–^, IgM^hi^/CD21/35^hi^; *N* = 5/group for Follicular, 3–4/group for germinal center B cells, 4–10 for marginal zone. **(D)** Total T1 transitional immature B cells per spleen at given time post-CLP. Gating: FSC/SSC, singlets, Live, CD19^+^/B220^+^, CD93^+^, IgM^hi^/CD23^–^; *N* = 3–4/group. **(E)** Total plasma cells per spleen at given time post-CLP. Gating: FSC/SSC, singlets, Live, CD19^+^/B220^+^, CD93^–^, B220^+^/CD138^+^; *N* = 3–4/group. **(F)** Total splenic CD4 T follicular helpers in the spleen at given time post-CLP. Gating: FSC/SSC, singlets, Live, CD90^+^/CD4^+^, PD1^+^/CXCR5^+^; *N* = 3–4/group.

Previous studies demonstrated that CLP depleted B cells via apoptosis (1, 15). The effects of CLP on specific B cell subsets, however, is unknown. Therefore, we examined splenic B cells obtained at baseline (T_0_) and at 24, 48, and 72 h post-CLP. Compared to unoperated (T_0_) controls, total splenic B cell numbers were significantly lower at 48 and 72 h post-CLP (Figure 1A). This difference was noted in both mature (CD93^–^, Figure 1B, left) and immature (CD93^+^, Figure 1B, right) B cells. Because the majority of B cells in the spleen were mature, these cells made the largest contribution to the reduction in total B cells.

Mature B cells can be divided into either follicular (FO, IgM^lo^/CD21/35^lo^) or marginal zone (MZ, IgM^hi^/CD21/35^hi^) B cells. Germinal center (FO GL7^+^) B cells are a subset of FO B cells that generate germinal centers and initiate mature antibody responses. Numbers of splenic FO, MZ and germinal center B cells were lower than T_0_ at 24, 48, and 72 h post-CLP (Figure 1C). FO B cells are further categorized as FO I B cells, which are resistant to depletion during infection or FO II B cells, which that transit to the spleen following B cell depletion to replenish both MZ and FO B cells. This decrease equally affected both FO I B cells that are more resistant to depletion during infection and FO II B cells (Supplementary Figure S4) (16, 17).

At 24, 48, and 72 h post-CLP, T1 (early emigrant) immature splenic B cells (that normally mature to form FO and MZ B cells) were depleted (Figure 1D). Numbers of splenic T2 and T3 immature B cells, which develop from T1 B cells during maturation, were not affected by CLP (data not shown) (18).

At 72 h post-CLP the number of plasma cells (mature B cells that can make functional antibody) was significantly increased; changes were not noted at earlier time points (Figure 1E).

T follicular helper cells (Tfh, CD90^+^/CD4^+^/PD1^+^/CXCR5^+^) interact with FO B cells to promote an effective B cell response, germinal center formation, and antibody production. At 24, 48, and 72 h following CLP the number of splenic CD4 Tfh was lower than at T_0_ (Figure 1F).

### Immune Education Attenuates the CLP-Induced Decrease in Memory Tfh Cells

**FIGURE 2:**
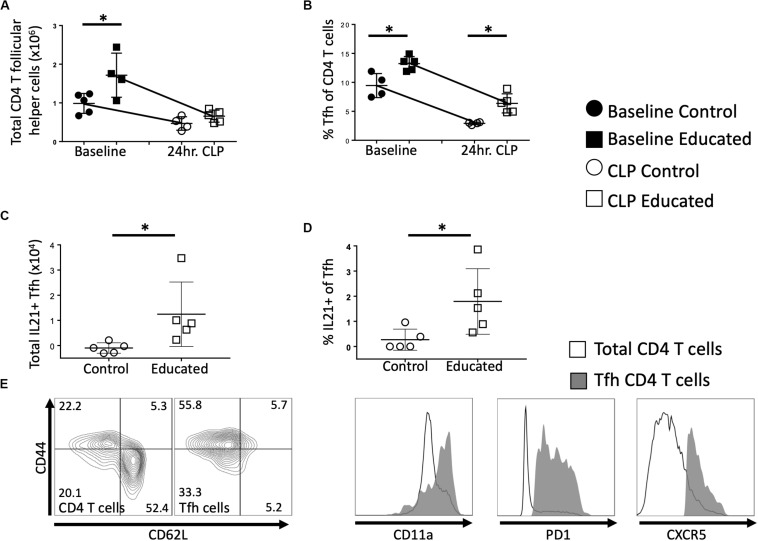
Effects of immune education on splenic CD4 T follicular helper cell response to CLP. C57Bl/6 laboratory mice were injected with anti-CD3ε or isotype control antibody. Thirty five days later mice were euthanized (T_0_) or subjected to CLP. Mice subjected to CLP were euthanized at 24 h and T follicular helper T cells were analyzed using flow cytometry. Each point represents results in an individual animal, central horizontal line indicates mean, upright and inverted Ts indicate standard deviation, data representative of two independent experiments. Filled circles – T_0_ in control mice; Filled square – T_0_ in immune educated mice; Open circle – 24 h post-CLP in control mice; Open square – 24 h post-CLP in immune educated mice. Data analyzed using two-way ANOVA with Sidak’s *post hoc* correction for multiple comparisons. *, significantly different from value in control mice at same time point; *p < 0.05. **(A)** Total number of CD4 T follicular helper cells per spleen. Gating: FSC/SSC, singlets, Live, CD90^+^/CD4^+^, PD1^+^/CXCR5^+^; *N* = 4–5/group. **(B)** Percentage of CD4 T cells with T follicular helper cell phenotype. Gating: FSC/SSC, singlets, Live, CD90^+^/CD4^+^, PD1^+^/CXCR5^+^; *N* = 4–5/group. **(C)** Total number of IL21-producing T follicular helper cells per spleen. Cells stimulated *ex vivo* with CD3/CD28 for 5 h in the presence of Brefeldin A. Numbers represent percent above background. Gating: FSC/SSC, singlets, Live, CD90^+^/CD4^+^, PD1^+^/CXCR5^+^, IL21^+^; *N* = 4–5/group. **(D)** Percentage of IL21 – producing T follicular helper cells per spleen. Cells stimulated *ex vivo* with CD3/CD28 for 5 h in the presence of Brefeldin A. Numbers represent percent above background. Gating: FSC/SSC, singlets, Live, CD90^+^/CD4^+^, PD1^+^/CXCR5^+^, IL21^+^; *N* = 4–5/group. **(E)** Flow cytometric plot demonstrating CD44/CD62L phenotype of Tfh compared to all CD4 T cells, along with CD11a, PD1, CXCR5 histograms. Line represents all CD4 T cells, Gray represents Tfh. Representative of 5 replicates.

We next examined the effects of immune education (induction of a diverse memory T cell repertoire using an anti-CD3ε activating antibody, as previously described) (11) on the Tfh and B cell responses to CLP. Relative to controls, immune education significantly increased the number (Figure 2A) and percentage (Figure 2B) of CD4 T cells expressing the Tfh phenotype. In both control and immune educated mice, CLP decreased the number of CD4 Tfh cells/spleen and the percentage of CD4 cells expressing the Tfh phenotypes. The CLP-induced decrease in the number of Tfh cell was significantly greater in educated than in control mice (0.9 × 10^6^ vs. 0.5 × 10^6^ cells/spleen, Figure 2A) but the percentage change in both groups was similar (approximately 7%, Figure 2B) and CLP did not induce a different change in Tfh in educated or control mice. Thus, both before and at 24 h post-CLP, Tfh cells represented a significantly greater proportion of CD4 T cells in educated mice than in controls.

Interleukin-21 (IL21) produced by Tfh cells interacts with FO B cells to promote differentiation and germinal center formation. In both educated and control baseline mice, no IL21 was detected following *ex vivo* T cell receptor (TCR; CD3/CD28) stimulation of Tfh cells. Similarly, TCR stimulation of Tfh cells isolated from control mice 24 h post-CLP did not elicit IL21 production (Figure 2C). In contrast, TCR-stimulation induced Tfh cells isolated from immune educated mice 24 h post-CLP to produce IL21 (Figure 2C) – that is, IL21 production was noted in approximately 2% of Tfh cells (Figure 2D). These Tfh cells were predominantly CD44^+^/CD11a^+^/CD62L^–^, consistent with a memory effector phenotype (Figure 2E).

### Immune Education Alters CLP-Induced Changes in Splenic Architecture

**FIGURE 3:**
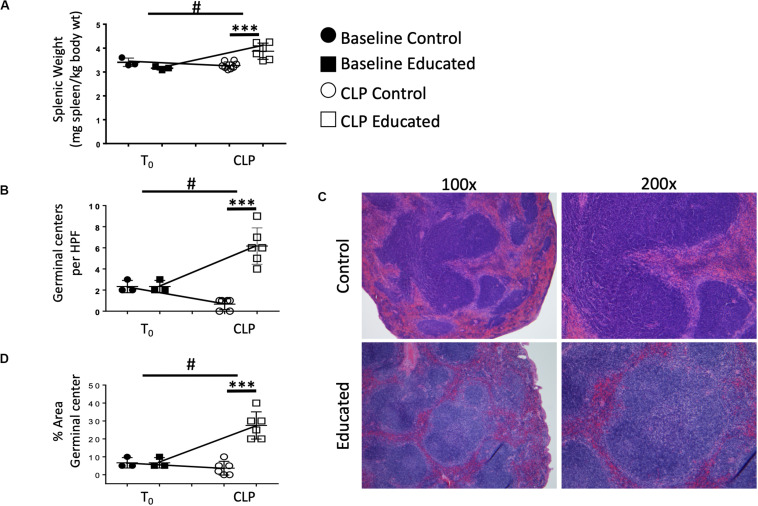
Effects of immune education splenic size and splenic germinal center formation 24 h post-CLP. C57Bl/6 laboratory mice underwent education or treatment with isotype control antibody. Thirty-five days later mice were euthanized (T_0_) or subjected to CLP. Mice subjected to CLP were euthanized 24 h later. Spleens were weighed immediately *ex vivo* and normalized to pre-CLP body weight. Spleens were fixed and stained with hematoxylin and eosin and analyzed for germinal center formation by blinded pathologists. Each point represents results in an individual animal, central horizontal line indicates mean, upright and inverted Ts indicate standard deviation, data representative of two independent experiments. Filled circles – T_0_ in control mice; Filled square – T_0_ in immune educated mice; Open circle – 24 h post-CLP in control mice; Open square – 24 h post-CLP in immune educated mice. Data analyzed using two-way ANOVA with Sidak’s *post hoc* correction for multiple comparisons. ***p < 0.001, significantly different from value in control mice at same time point; ^#^*p* < 0.05, slope of line connecting T0 mean and mean 24 h post-CLP significantly different than slope of line for control mice. **(A)** Splenic weight normalized to pre-CLP body weight. *N* = 3/group. **(B)** Germinal centers per high power field. *N* = 6/group. **(C)** Hematoxylin and eosin stain of the spleen visualized at 100× or 200× revealing germinal centers. Representative of 6 slides each. **(D)** Percent area covered by germinal centers in the spleen. *N* = 6/group.

When activated, Tfh cells promote a follicular B cell response and germinal center formation. Therefore, we examined the effects of the immune education–induced increase in Tfh cells on splenic architecture 24 h following CLP. Neither education alone nor CLP in control mice altered the weight of the spleen (Figure 3A). Relative to both baseline in educated mice prior to CLP and to post-CLP controls, CLP in educated mice increased splenic weight by 25% (Figure 3A). The CLP-induced splenomegaly in educated mice was associated with an increased number of germinal centers (Figure 3B) and an increase in the area of the spleen taken up by germinal centers (Figures 3C,D and Supplementary Figure S5).

### Immune Education Increases CLP Induced FO B Cell Responses in the Spleen

**FIGURE 4:**
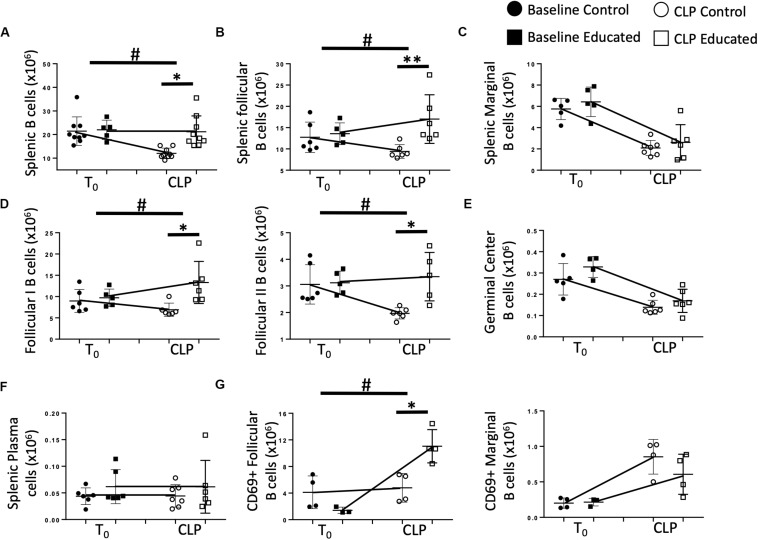
Effects of immune education on B cell populations 24 h post-CLP. C57Bl/6 laboratory mice underwent education or treatment with isotype control antibody. Thirty-five days later mice were euthanized (T_0_) or subjected to CLP. Mice subjected to CLP were euthanized 24 h later. Spleens were homogenized and B cell populations were analyzed using flow cytometry. Each point represents results in an individual animal, central horizontal line indicates mean, upright and inverted Ts indicate standard deviation, data representative of two independent experiments. Filled circles – T_0_ in control mice; Filled square – T_0_ in immune educated mice; Open circle – 24 h post -CLP in control mice; Open square – 24 h post-CLP in immune educated mice. Data analyzed using two-way ANOVA with Sidak’s *post hoc* correction for multiple comparisons. *p < 0.05, significantly different from value in control mice at same time point; ^#^*p* < 0.05, slope of line connecting mean at T_0_ to mean value 24 h post-CLP significantly different than same line in control mice. **(A)** Total splenic B cells. Gating: FSC/SSC, singlets, Live, CD19^+^/B220^+^; *N* = 5–9/group. **(B)** Total follicular (Left) and marginal zone (Right) B cells per spleen. Gating: Follicular B cells: FSC/SSC, singlets, Live, CD19^+^/B220^+^, CD93^–^, B220^+^/CD138^–^, IgM^lo^/CD21/35^lo^. *N* = 5–6/group. **(C)** Total marginal zone B cells per spleen. Gating: Marginal zone: FSC/SSC, singlets, Live, CD19^+^/B220^+^, CD93^–^, B220^+^/CD138^–^, IgM^hi^/CD21/35^hi^. *N* = 5–6/group. **(D)** Total follicular I (left) and follicular II (right) B cells per spleen. Gating: Follicular I B cells: FSC/SSC, singlets, Live, CD19^+^/B220^+^, CD93^–^, B220^+^/CD138^–^, IgM^lo^/CD21/35^lo^, IgD^+^/IgM^lo^. Follicular II B cells: FSC/SSC, singlets, Live, CD19^+^/B220^+^, CD93^–^, B220^+^/CD138^–^, IgM^lo^/CD21/35^lo^, IgD^+^/IgM^mid^ *N* = 5–6/group. **(E)** Total germinal center B cells per spleen. Gating: FSC/SSC, singlets, Live, CD19^+^/B220^+^, CD93^–^, B220^+^/CD138^–^, IgM^lo^/CD21/35^lo^, GL7^+^; *N* = 5–6/group. **(F)** Total plasma cells per spleen. Gating: FSC/SSC, singlets, Live, CD19^+^/B220^+^, CD93^–^, B220^+^/CD138^+^; *N* = 7/group. **(G)** Total CD69 + follicular (left) and CD69 + marginal (right) B cells per spleen. Gating: Follicular B cells: FSC/SSC, singlets, Live, CD19^+^/B220^+^, CD93^–^, B220^+^/CD138^–^, IgM^lo^/CD21/35^lo^, CD69^+^/IgM^+^. Marginal B cells: FSC/SSC, singlets, Live, CD19^+^/B220^+^, CD93^–^, B220^+^/CD138^–^, IgM^hi^/CD21/35^hi^, CD69^+^/IgM^+^ *N* = 3–4/group.

We next examined the effects of immune education on CLP-induced changes in splenic B cell phenotypes detailed in Figure 1. T cell education had minimal effect on B cells prior to CLP (Figure 4A). In contrast to the reduction in total B cells observed 24 h post-CLP in control mice (Figures 1A, 4A), CLP did not alter total B cell numbers in immune educated mice (Figure 4A). Similarly, while CLP in control mice reduced the number of FO B cells (Figure 1C), no such change was noted 24 h post-CLP in educated mice (Figure 4B). In contrast, immune education did not alter the CLP-induced decrease in MZ B cells (Figures 1C, 4C). While the number of FO I B cells in control animals was not changed, in immune educated mice the number of FO I cells present 24 h post-CLP was greater than in control mice (Figure 4D). However, CLP reduced the number of FO II B cells in controls but not in educated mice (Figure 4D). The combined effects of CLP on FO I and FO II B cells accounted for the overall difference in the response of FO B cells observed 24 h post-CLP in educated mice (Figures 4B,D). The number of germinal center B cells in both control and educated mice was equally reduced 24 h post-CLP (Figure 4E) while neither CLP nor immune education affected the number of splenic plasma cells (Figure 4F).

B cell activation through antigen recognition is associated with upregulation of the surface marker CD69 (19). T cell education had no effect on the number of FO or MZ B cells expressing CD69 prior to CLP (Figure 4G). Following CLP, the number of CD69^+^ FO B cells in control mice was not altered. In contrast, CLP induced an increase in CD69^+^ FO B cells in educated mice, indicating increased activation (Figure 4G). When MZ B cells were examined for CD69 expression, CLP induced a similar increase in CD69^+^ MZ B cells compared to baseline numbers in baseline mice, but immune education had no effect on activation of these cells (Figure 4G).

### CD4 T Cell Memory Causes Persistent Alteration in the B Cell Response 60 Days Post-CLP

**FIGURE 5:**
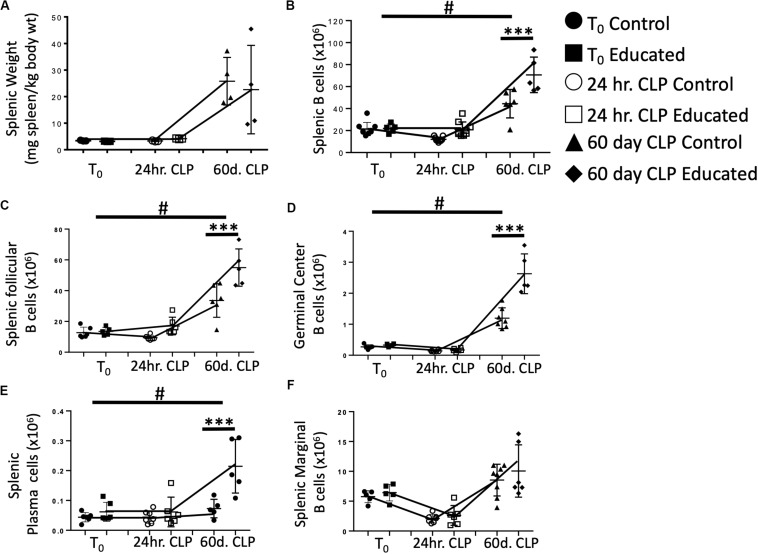
Effects of immune education on Splenic Weight and B cell populations at T_0_, 24 h and 60 days post-CLP. C57Bl/6 laboratory mice underwent education or treatment with isotype control antibody. Thirty-five days later mice were euthanized (T_0_) or subjected to CLP. Mice subjected to CLP were euthanized 24 h (24 h CLP) or 60 days (60 days CLP) later. Spleens were weighed and homogenized and B cell populations were analyzed using flow cytometry. Each point represents results in an individual animal, central horizontal line indicates mean, upright and inverted Ts indicate standard deviation, data representative of two independent experiments. Filled circles – T_0_ in control mice; Filled square – T_0_ in immune educated mice; Open circle – 24 h post-CLP in control mice; Open square – 24 h post-CLP in immune educated mice. Data analyzed using two-way ANOVA with Sidak’s *post hoc* correction for multiple comparisons. #, slope of line connecting T_0_ mean and mean 24 h post-CLP significantly different than slope of line for control mice. ***p < 0.001, significantly different from value in control mice at same time point; ^#^*p* < 0.05, slope of line connecting mean at T_0_ to mean value 24 h post-CLP significantly different than same line in control mice. **(A)** Splenic weight normalized to pre-CLP body weight. *N* = 4/group. **(B)** Total splenic B cells. Gating: FSC/SSC, singlets, Live, CD19^+^/B220^+^; *N* = 3–4/group. **(C)** Total follicular B cells. Gating: Follicular B cells: FSC/SSC, singlets, Live, CD19^+^/B220^+^, CD93^–^, B220^+^/CD138^–^, IgM^lo^/CD21/35^lo^; *N* = 4/group. **(D)** Total germinal center B cells. Gating: FSC/SSC, singlets, Live, CD19^+^/B220^+^, CD93^–^, B220^+^/CD138^–^, IgM^lo^/CD21/35^lo^, GL7^+^; *N* = 4/group. **(E)** Total plasma cells. Gating: FSC/SSC, singlets, Live, CD19^+^/B220^+^, CD93^–^, B220^+^/CD138^+^; *N* = 4/group. **(F)** Total marginal zone B cells. Gating: FSC/SSC, singlets, Live, CD19^+^/B220^+^, CD93^–^, B220^+^/CD138^–^, IgM^hi^/CD21/35^hi^; *N* = 4/group.

Sjaastad et al. have demonstrated that CLP attenuated the Tfh and FO B cell response to specific antigens (10). This decreased response persisted for at least 30 days following CLP. We have shown that immune education induced prior to CLP altered the acute response to CLP by (1) increasing the percentage of CD4 T cells expressing the Tfh phenotype (Figure 2B), (2) increasing IL21 production (Figures 2C,D) by Tfh cells, (3) increasing germinal center formation (Figures 3B,C), and increasing the number of splenic follicular B cells (Figure 4B). The effects of immune education, and specifically of the pre-existing presence of a substantial number of Tfh cells, on the long-term immune response to CLP is unknown. We therefore examined B cell responses 60 days after CLP in control and immune educated mice. Results are detailed in Figure 5. At this more remote time point, splenic weight in both control and immune educated mice was similarly increased (Figure 5A). The total number of splenic B cells increased relative to T_0_ and 24 h post-CLP numbers but the increase in educated mice was significantly greater than in controls (Figure 5B). A similar response was noted in splenic follicular (Figure 5C) and germinal center cells (Figure 5D) as well as in splenic plasma cells cell (Figure 5E). Marginal B cells were increased relative to T_0_ and 24 h post-CLP numbers but, as seen in Figure 4C, there was no difference in marginal B cells between educated and control mice at 60 days (Figure 5F).

### Education Increases the Antigen-Specific B Cell Response to CLP

**FIGURE 6:**
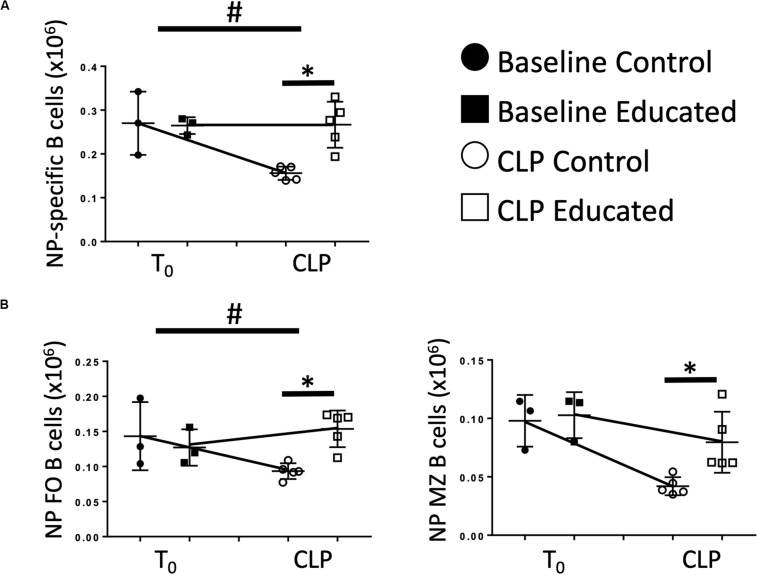
Effects of immune education on NP-specific B cell populations. C57Bl/6 laboratory mice underwent immune education or treatment with isotype control antibody. Thirty-five days later were treated with intraperitoneal 4-hydroxy-3-nitrophenylacetic acid (NP), 5 μg suspended in PBS (T_0_) or subjected to CLP and treated with NP (7 days CLP). Mice were euthanized 7 days later. Spleens were homogenized and B cell populations were analyzed using flow cytometry. Each point represents results in an individual animal, central horizontal line indicates mean, upright and inverted Ts indicate standard deviation, data representative of two independent experiments. Filled circles – T_0_ in control mice; Filled square – T_0_ in immune educated mice; Open circle – 24 h post-CLP in control mice; Open square – 24 h post-CLP in immune educated mice. Data analyzed using two-way ANOVA with Sidak’s *post hoc* correction for multiple comparisons. *p < 0.05, significantly different from value in control mice at same time point; ^#^*p* < 0.05, slope of line connecting T_0_ mean and mean 24 h post-CLP significantly different than slope of line for control mice. **(A)** Total splenic NP-specific B cells per spleen. Gating: FSC/SSC, singlets, Live, CD19^+^/B220^+^, CD19^+^/NP^+^; *N* = 5–9/group. **(B)** Total NP-specific follicular (Left) and marginal zone (Right) B cells per spleen. Gating: Follicular B cells: FSC/SSC, singlets, Live, CD19^+^/B220^+^, CD19^+^/NP^+^, CD93^–^, B220^+^/CD138^–^, IgM^lo^/CD21/35^lo^, Marginal Zone B cells FSC/SSC, singlets, Live, CD19^+^/B220^+^, CD19^+^/NP^+^, CD93^–^, B220^+^/CD138^–^, IgM^hi^/CD21/35^hi^; *N* = 5–6/group.

Immune education induced general changes to the B cell response to CLP (Figure 4). However, the effects of CLP on specific B cell responses to known antigen present at the time of CLP in the presence of educated Tfh are unknown. Therefore, we administered 5 μg 4-hydroxy-3-nitrophenylacetic acid or vehicle into the peritoneum of control and educated mice at the time of CLP to mimic antigen introduction at the time of insult. Results were compared to what was observed 7 days after a similar injection was given to unoperated mice. Compared to mice administered NP but not subjected to CLP, NP-specific B cells could not be detected in animals not exposed to NP. Seven days following CLP, the number of NP-specific B cells in control mice was significantly lower than that measured in control animals subjected to injection only (Figure 6A). In contrast, the number of NP-specific B cells was maintained in educated mice following CLP. A similar result was noted in NP-specific FO B cells (Figure 6B, left), while NP-specific MZ B cells were not maintained in educated mice (Figure 6B, right).

## Discussion

The data presented here examine the B cell response to CLP in a murine model that includes a broad repertoire of memory T cells (“immune educated mice”). This particular component of adaptive immunity is not present when CLP is performed on standard laboratory (control) mice (7), and constitutes an important deficiency in this most commonly – used model of human sepsis (3, 4). Our data demonstrate that the presence of T cell memory altered several aspects of the acute response to CLP, most notably increasing induced IL21 production by Tfh cells indicating increased functionality. Increased functionality, in turn, reversed the CLP-induced decrease in splenic B cell populations noted in control animals, enhancing FO B cell and germinal center development. Further, B cell activation, as indicated by CD69 expression, was increased in the presence of T cell memory. While CLP diminished the response to a specific antigen in control mice, the response was preserved in immune educated animals. This finding indicates that memory Tfh cells may be required for antigen-specific responses in the presence of inflammation. Most importantly, the effects of immune education on B cell maturation were still present 60 days after CLP. Thus, a mature B cell response may contribute to differences in both short – and long – term abnormalities between control and immune educated mice. Further, the results suggest that T cell memory, in part via its effect on B cell development, plays an important role in the pathogenesis of human sepsis.

Little is known concerning the effects of preexisting T cell memory on the acute response to CLP. Tfh assist FO B cells in converting from short-lived, naïve IgM^+^/IgD^+^ B cells to long-lived, memory B cells and plasma cells that can efficiently produce antibodies to assist the body in preventing recurrent infection. FO B cells that receive Tfh assistance are able to induce antibody class-switching with increased antibody affinity to both extracellular and intracellular pathogenic antigens. Without T cell help, only low affinity antibodies can be produced and class-switching is limited. Considering that most adult humans have a significant Tfh compartment prior to a septic event, the effect of pre-existing Tfh on the B cell response must be considered. Recent clinical data supports that decreased circulating Tfh at sepsis onset in human subjects correlates with decreased B cell maturation during sepsis and decreased survival (2).

Murine CLP has long been the animal model of choice for human sepsis (3, 4). There are, however, two commonly voiced concerns about this approach. First, based on immunologic and genetic differences, some have opined that differences between CLP and human sepsis are too profound for translation of findings from mice to men (5). Second, improved acute care has identified a cohort of sepsis survivors who have significant long-term health problems. To date, murine equivalents of these late or persistent abnormalities have not been well-characterized or investigated. The data presented here is pertinent to both concerns, emphasizing that addressing the first issue is required to address the second.

In previous work we have used an anti-CD3ε antibody to induce a broad repertoire of memory T cells in C57Bl/6 mice (11). The result has been a significant change in the response to CLP (Taylor et al., unpublished data). The findings presented here further characterize the role of T cell memory in the response to CLP. Specifically, our findings demonstrate that the presence of memory Tfh cells, which has not been examined in either CLP or in human sepsis, is an essential component in the activation of B cells responses. These findings may have direct clinical relevance. Many years ago, Meakins et al., observed that a failure to resolve anergy was a poor prognostic factor in septic surgical and trauma patients (20). It is difficult to assess the ability of B cells to form antibody following an inflammatory insult. However, our findings indicate that an inadequate Tfh response limits B cell receptor signaling and maturation. One potential consequence of a lack of Tfh cells would be an acceleration of B cell apoptosis (21, 22), a finding present at autopsy in patients with fatal sepsis (1). Supporting this explanation is a recent prospective cohort study of sepsis patients indicating activation-associated cell death is a major driver of B cell lymphopenia in sepsis; preexisting Tfh may increase the apoptotic threshold of activated B cells, partially rescuing the B cell response (23).

Our data indicate that the acute CLP-induced decrease in mature B cell numbers was reversed 60 days following the procedure. The fact that this increase was more greater in immune educated mice likely reflects increased functionality by memory Tfh cells.

Our findings may also have relevance to what has been viewed as a major limitation of CLP – the use of laboratory mice (5, 6). Translation of results from CLP to human sepsis has been poor. It has been noted that, in contrast to humans, lab mice lack a memory T cell compartment (7, 8). Our group has developed a method of inducing widespread T cell memory through administration of an anti-CD3ε activating antibody. This “education” leads to widespread CD4 and CD8 T cell memory induction. This approach altered the response to CLP in a manner that enhances features consistent with sepsis – a more pronounced innate immune response, more profound organ dysfunction and decreased survival (Taylor, et al., unpublished data). The importance of the effects of immune education on CLP-induced changes in B cell numbers is unclear. However, it reverses acute depression of B cell responses that follows CLP. The augmentation of this early response to CLP indicates that perhaps a major component of the early response to sepsis has been neglected and that T cell memory may have important effects on the long-term responses to CLP and sepsis.

More importantly, the remaining B cells in the spleen that are not eliminated during the sepsis response, while low in number, may be mounting an effective antibody response that could play an important role in protective immunity. When examined, there was no difference in total IgG levels in the serum of mice at any time point (data not shown), indicating that the immune response at the cellular level may not be reflected in changes in total immunoglobulin repertoire, but instead, may represent alteration in specific B cell clones as demonstrated by an NP-specific B cell response with introduction of antigen. Addition of Tfh may help refine the B cell response and allow for antibody recognition of different epitopes that cannot be recognized without T cell help.

The Tfh memory response is understudied in CLP and could be an important target in future treatment of sepsis as a way to modulate the B cell response in a more effective manner. Tfh modulation may represent a target for preventing post-sepsis immunosuppression in the future.

## Data Availability Statement

The raw data supporting the conclusions of this article will be made available by the authors, without undue reservation.

## Ethics Statement

The animal study was reviewed and approved by Institutional Animal Care and Use Committee of the Feinstein Institutes for Medical Research of Northwell Health.

## Author Contributions

MT performed all experiments, analyzed the data, and wrote the manuscript. MB, AN-K, and MA assisted in cecal ligation, puncture procedure, animal handling, data analysis, and writing the manuscript. YY and KR performed histological slide preparation and analysis, and assisted in writing the manuscript. BB assisted in experimental design, analysis of data, and writing. CD supervised and assisted in experimental design, analysis of data, and writing. All authors contributed to the article and approved the submitted version.

## Conflict of Interest

CD is a consultant for Enlivex Therapeutics Inc, Jerusalem, Israel. The remaining authors declare that the research was conducted in the absence of any commercial or financial relationships that could be construed as a potential conflict of interest.

## Supplementary Material

The Supplementaryx Material for this article can be found online at: https://www.frontiersin.org/articles/10.3389/fimmu.2020.01946/full#supplementary-material

FIGURE S1 Murine Sepsis Score comparing control and immune educated mice following CLP. C57Bl/6 laboratory mice underwent education or treatment with isotype control antibody. Thirty-five days later mice were subjected to CLP and monitored daily for clinical scoring using the murine sepsis score. Data as mean ± standard deviation, ^∗^*p* < 0.001 for treatment effect over time by mixed effects modeling. Representative of two independent experiments. *N* = 5/group.

FIGURE S2 Gating Strategy for Tfh cells. Splenic T cell populations shown from educated mouse 24 h following CLP without TCR stimulation for all except cytokine staining, which is shown following TCR stimulation.

FIGURE S3 Gating Strategy for B cells. Splenic B cell populations shown from educated mouse 24 h following CLP (18).

FIGURE S4 Effects of CLP on total B cells and B cell subtypes in the spleen. C57Bl/6 laboratory mice underwent CLP and were euthanized at given timepoints. Data obtained using flow cytometry. Unmanipulated mice were used as T_0_ controls. Data as mean ± standard deviation, ^∗^*p* < 0.05, ^∗∗^*p* < 0.01 for spleen compared to T_0_ by one-way ANOVA with Dunnett correction for multiple comparisons. Total follicular I (left) and follicular II (right) B cells per spleen at given time post-CLP. Gating: Follicular I B cells: FSC/SSC, singlets, Live, CD19^+^/B220^+^, CD93^–^, B220^+^/CD138^–^, IgM^lo^/CD21/35^lo^, IgD^+^/IgM^lo^. Follicular II B cells: FSC/SSC, singlets, Live, CD19^+^/B220^+^, CD93^–^, B220^+^/CD138^–^, IgM^lo^/CD21/35^lo^, IgD^+^/IgM^mid^ *N* = 3–4/group.

FIGURE S5 Effects of immune education on splenic germinal center formation in the spleen following CLP. C57Bl/6 laboratory mice underwent CLP and were euthanized at 24 h. Spleens were fixed and stained with hematoxylin and eosin and analyzed for germinal center formation by blinded pathologists. Photos are representative of two independent experiments. **(A)** Germinal center as indicated by red circle with central paling in white pulp of spleen. **(B)** Hematoxylin and eosin stain of the spleen in control and educated mice 40× magnification. Representative of 6 slides each.

TABLE S1 Antibodies used for this manuscript.
