# Supplementary figures and images for: CD4 T Follicular Helper Cells Prevent Depletion of Follicular B Cells in Response to Cecal Ligation and Puncture
Source: Front Immunol. 2020 Aug 12;11:1946. doi: 10.3389/fimmu.2020.01946 (PMC7434988; doi:10.3389/fimmu.2020.01946)

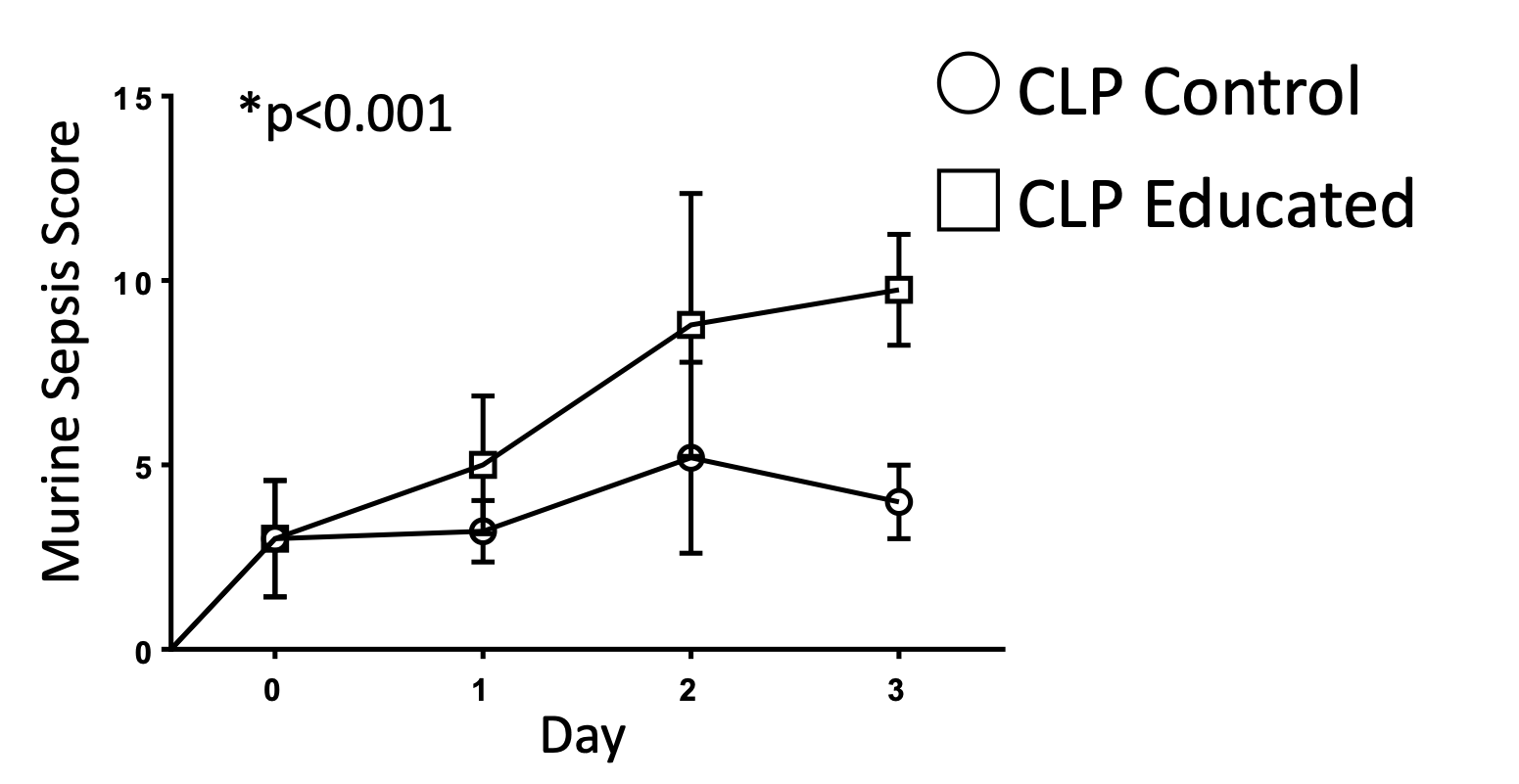

Supplement: FIGURE S1 — Murine Sepsis Score comparing control and immune educated mice following CLP. C57Bl/6 laboratory mice underwent education or treatment with isotype control antibody. Thirty-five days later mice were subjected to CLP and monitored daily for clinical scoring using the murine sepsis score. Data as mean ± standard deviation, ∗p < 0.001 for treatment effect over time by mixed effects modeling. Representative of two independent experiments. N = 5/group. [file Image_1.TIFF]

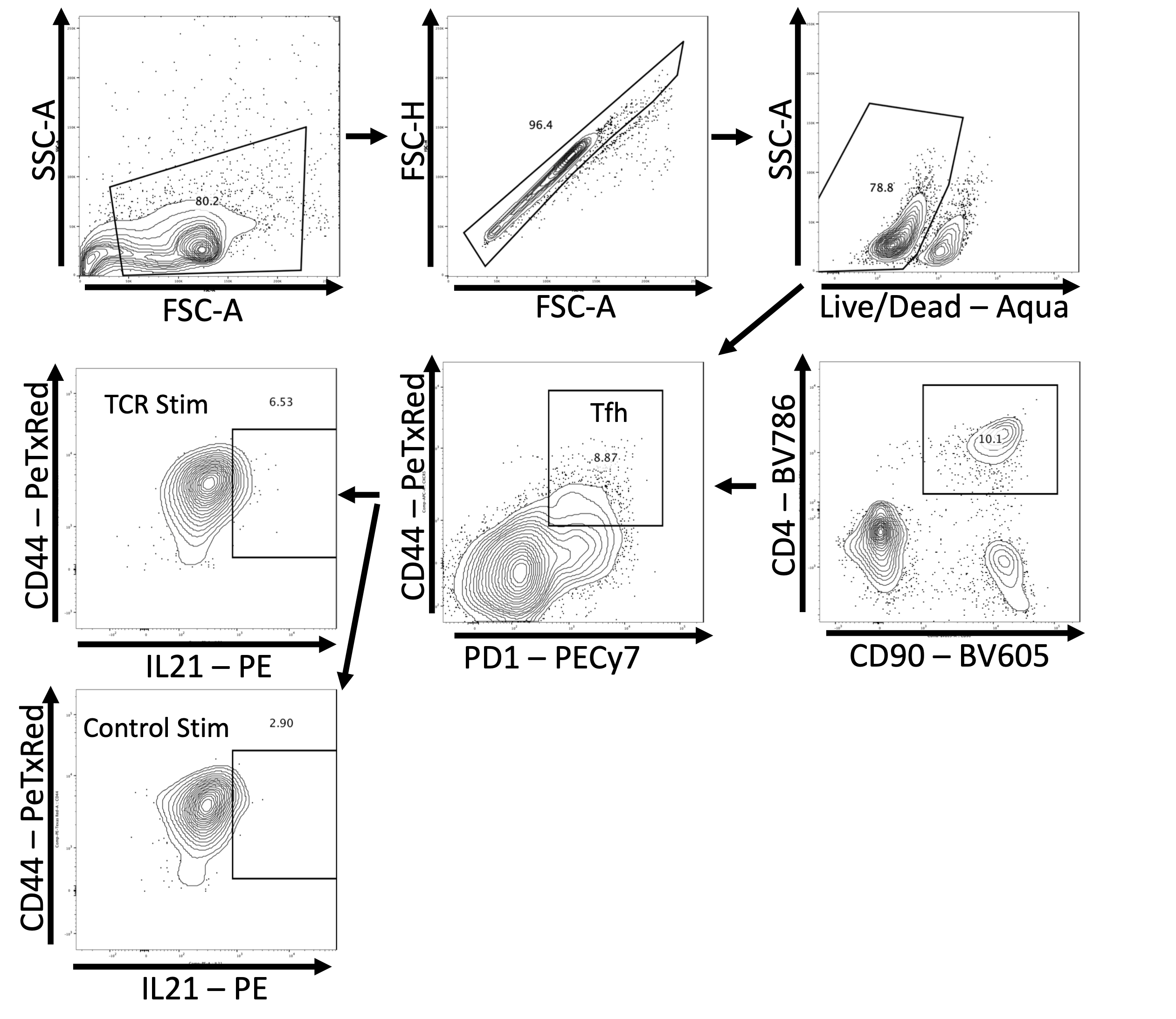

Supplement: FIGURE S2 — Gating Strategy for Tfh cells. Splenic T cell populations shown from educated mouse 24 h following CLP without TCR stimulation for all except cytokine staining, which is shown following TCR stimulation. [file Image_2.TIFF]

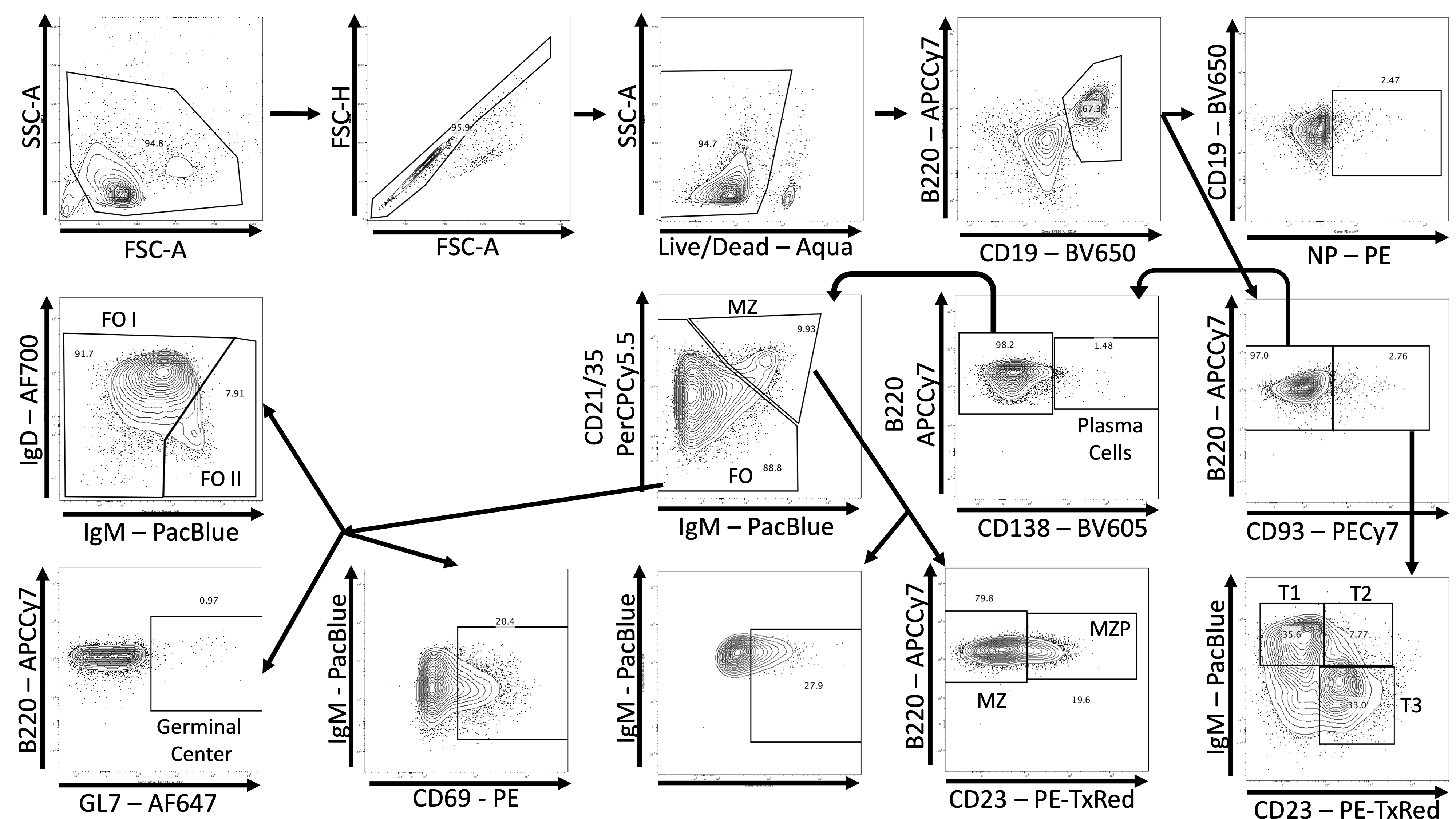

Supplement: FIGURE S3 — Gating Strategy for B cells. Splenic B cell populations shown from educated mouse 24 h following CLP (18). [file Image_3.TIFF]

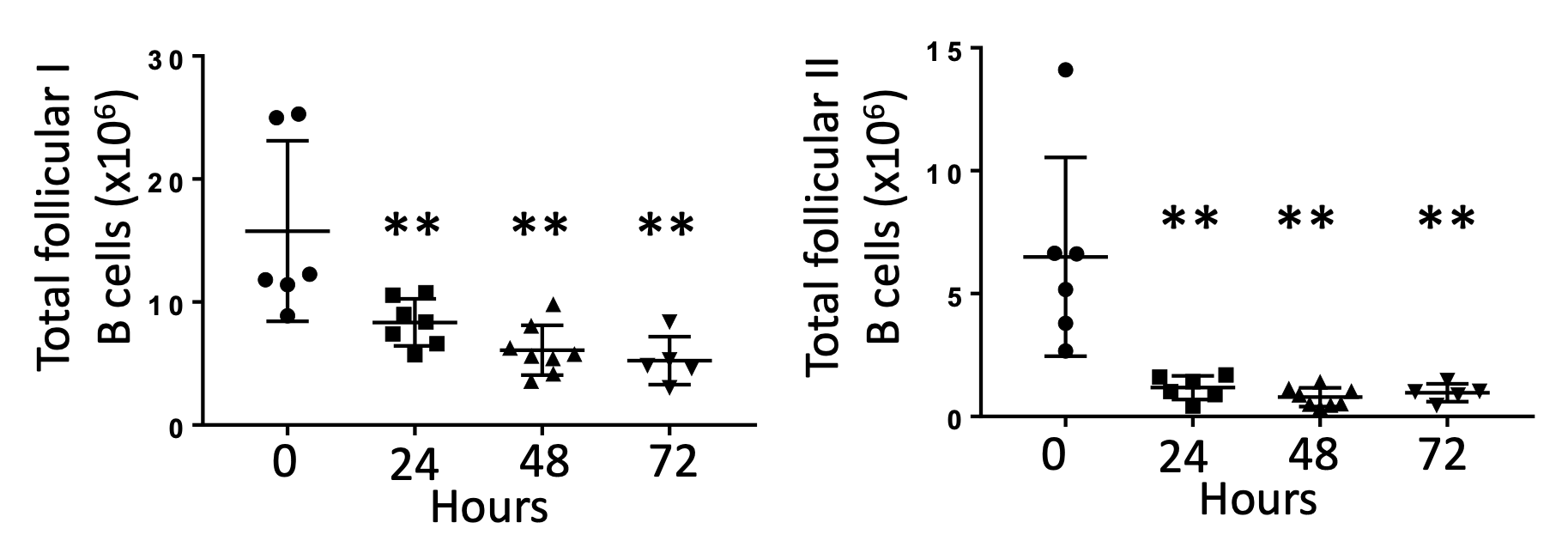

Supplement: FIGURE S4 — Effects of CLP on total B cells and B cell subtypes in the spleen. C57Bl/6 laboratory mice underwent CLP and were euthanized at given timepoints. Data obtained using flow cytometry. Unmanipulated mice were used as T0 controls. Data as mean ± standard deviation, ∗p < 0.05, ∗∗p < 0.01 for spleen compared to T0 by one-way ANOVA with Dunnett correction for multiple comparisons. Total follicular I (left) and follicular II (right) B cells per spleen at given time post-CLP. Gating: Follicular I B cells: FSC/SSC, singlets, Live, CD19+/B220+, CD93–, B220+/CD138–, IgMlo/CD21/35lo, IgD+/IgMlo. Follicular II B cells: FSC/SSC, singlets, Live, CD19+/B220+, CD93–, B220+/CD138–, IgMlo/CD21/35lo, IgD+/IgMmid N = 3–4/group. [file Image_4.TIFF]

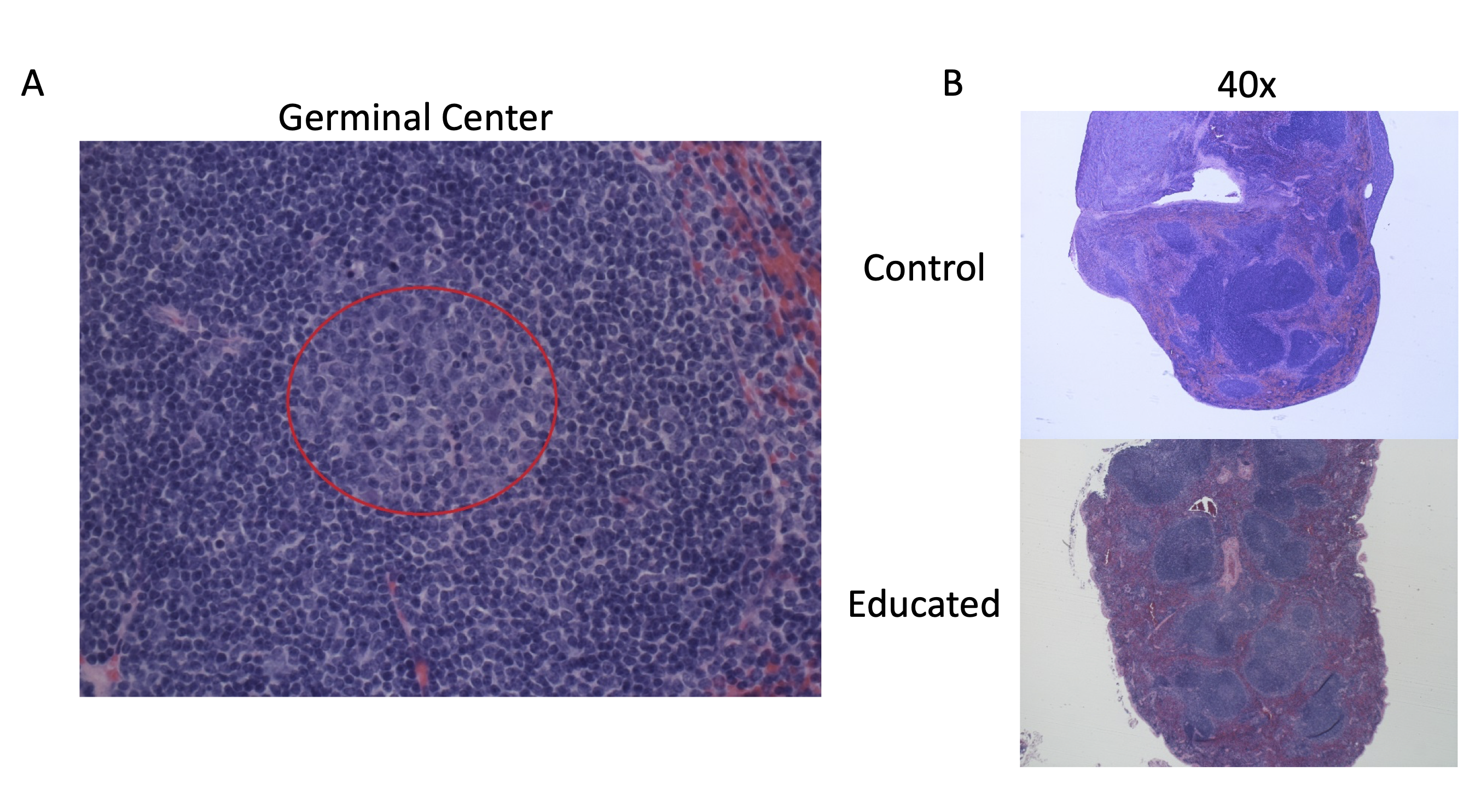

Supplement: FIGURE S5 — Effects of immune education on splenic germinal center formation in the spleen following CLP. C57Bl/6 laboratory mice underwent CLP and were euthanized at 24 h. Spleens were fixed and stained with hematoxylin and eosin and analyzed for germinal center formation by blinded pathologists. Photos are representative of two independent experiments. (A) Germinal center as indicated by red circle with central paling in white pulp of spleen. (B) Hematoxylin and eosin stain of the spleen in control and educated mice 40× magnification. Representative of 6 slides each. [file Image_5.TIFF]
