# Supplementary material for: CD4 T Follicular Helper Cells Prevent Depletion of Follicular B Cells in Response to Cecal Ligation and Puncture
Source: Front Immunol. 2020 Aug 12;11:1946. doi: 10.3389/fimmu.2020.01946 (PMC7434988; doi:10.3389/fimmu.2020.01946)
Supplement: TABLE S1 — Antibodies used for this manuscript. [file Table_1.pdf]

Target	Clone	Catalogue Number	Company	Fluorophore	Host Species
CD90.2 (Thy1.2)	53-2.1	140318	Biolegend	BV605	Rat
CD44	IM7	61-0441082	eBioscience	PE-eFluor610	Rat
CD4	RM4-5	563727	BD Horizon	BV786	Rat
CD62L	MEL-14	564108	BD Bioscience	BV650	Rat
CD11a	M17/a	740676	BD Bioscience	BV711	Rat
PD-1 (CD279)	29F.1A12	135215	Biolegend	PE/Cy7	Rat
CXCR5	L138D7	145532	Biolegend	AF647	Rat
CD45R/B220	RA3-6B2	103224	Biolegend	APC/Cy7	Rat
CD19	6D5	115541	Biolegend	BV650	Rat
CD19	6D5	115543	Biolegend	BV785	Rat
CD138	281-2	142515	Biolegend	BV605	Rat
CD93	AA4.1	136505	Biolegend	PE/Cy7	Rat
IgM	RMM-1	406517	Biolegend	BV421	Rat
IgD	11-26c-2a	405729	Biolegend	AF700	Rat
GL7	GL7	144605	Biolegend	AF647	Rat
CD23	B3B4	101633	Biolegend	PE/Dazzle594	Rat
CD21/35	7E9	123415	Biolegend	PerCPCy5.5	Rat
IL21	MHALX21	12-7213-80	eBioscience	PE	Rat
CD69	H1.2F3	104508	Biolegend	PE	Hamster
NP-PE		N-5070-1	Biosearch Tech	PE	
4-Hydroxy-3-nitrophenylacetic acid		219924-1G	SigmaAldrich		
In Vivo					
Ultra-LEAF Purified anti-Mouse CD3e	145-2C11	100359	Biolegend		Hamster
Ultra-LEAF Purified Armenian Hamster IgG Isotype Ctrl	HTK888	400959	Biolegend		Hamster
